# Understanding Dynamics: A Systematic Review of the Attitudes, Knowledge, and Competencies of European Frontline Professionals Toward Domestic Abuse

**DOI:** 10.1177/15248380251344311

**Published:** 2025-06-25

**Authors:** Rosalyn Millar, Olivia Crawford, Cherie Armour, Katrina McLaughlin

**Affiliations:** 1 Queen’s University, Belfast, UK

**Keywords:** domestic abuse, attitudes, professionals, medical staff, law practitioners, police

## Abstract

There remains a paucity of European research on attitudes and responses to domestic abuse from frontline workers and professionals who regularly encounter domestic abuse or engage with domestic abuse legislation. This systematic review synthesized qualitative, quantitative, and mixed-method peer-reviewed studies that explored professionals’ knowledge, attitudes, and competencies related to domestic abuse. The professionals included medical staff (doctors, nurses, midwives), social care professionals, police officers, and criminal justice practitioners. The review was conducted on current European studies published between 2014 and 2025 and was reported in accordance with the Preferred Reporting Items for Systematic Reviews and Meta-Analyses (PRISMA) guidelines. Databases consulted included APA PsycInfo, Scopus, Web of Science, PubMed, Sociological Abstracts, International Bibliography of the Social Sciences, Social Services Abstracts, and Google Scholar. Full-text review was performed on 273 articles, of which 36 were deemed appropriate for inclusion. The review included 8 multi-country studies spanning the United Kingdom (England, Scotland, and Wales), and 28 single-country studies conducted in England, Sweden, Turkey, Portugal, the Republic of Ireland, Wales, Bosnia, Spain, Italy, Slovenia, and Hungary. A narrative and thematic synthesis categorized professional attitudes toward domestic abuse into four emergent themes: attitudes toward engagement and responsibility, attitudes toward victims, knowledge and understanding of domestic abuse, and attitudes as predictors of professional practice. This review addresses a dearth of research and provides recommendations for promoting proactive practice among professionals most likely to receive disclosures of abuse.

## Introduction

Significant progress has been made in addressing domestic abuse across Europe, notably with the introduction of the Istanbul Convention in 2014. This Convention recognizes domestic abuse and gender-based violence as human rights violations and forms of discrimination, as defined by the 1979 United Nations Convention (Council of Europe, 2024). The European Union (EU) signed the Convention in 2017 and completed its accession in 2023, demonstrating its commitment to combating gender-based violence, particularly in relation to EU institutions, judicial cooperation, asylum, and non-refoulement (Council of Europe, 2024). The global cost of domestic abuse is estimated to be approximately 2% of global gross domestic product. In 2016, this cost was $1.5 trillion, and in 2025, it is projected to reach $2.3 trillion (O’Neill, 2023; UN Women, 2020). Some of these costs are linked to the loss of economic productivity within healthcare systems, as victims often suffer from physical injuries, mental health issues, and chronic health problems. Additional economic impacts extend to the criminal justice system, which incurs costs beyond those faced by the victim. These include expenses related to police investigations, legal proceedings, the incarceration of perpetrators, and the management of long-term effects, such as repeat offenses (Rothman et al., 2007).

The World Health Organization (2024) estimates that globally, one in three women will experience sexual, physical, or non-physical intimate partner violence in their lifetime. In Europe, there is a considerable lack of publicly available data on EU-wide abuse statistics. The most recent data from 2021 indicates that gender-based violence is most prevalent in Finland, Sweden, Hungary, Denmark, and Luxembourg, where over 45% of women reported experiencing physical violence (including threats) or sexual violence during adulthood by any perpetrator. These statistics are consistent for psychological violence as well, with Hungary, Romania, and Finland experiencing the highest rates (Eurostat, 2024a). Countries such as Germany, Romania, and Czechia reported that more than 45% of victims were unaware of the supports available to them, with these findings also strongly correlated with a lack of awareness about legal aid (Eurostat, 2024b, 2024c).

This lack of awareness can be particularly detrimental when victims are at their most vulnerable. Leaving an abusive relationship or challenging the perpetrator legally is one of the most dangerous times for victims and is often when they are at the highest risk of domestic homicide (Humphreys & Thiara, 2003). It is well documented that the impact of legal proceedings on victims can vary significantly depending on the quality of professional responses received (Saxton et al., 2021). Effective professional practice is characterized by listening to victims, providing empathetic, trauma-informed support, offering clear information, and ensuring access to resources (McKenna & Holtfreter, 2021). Such practices can help reduce psychological harm and facilitate recovery (Y. C. Chu et al., 2024). In contrast, poor practice typically involves professionals dismissing, discrediting, or minimizing the victim’s experiences, which can lead to secondary retraumatization and further psychological harm (Epstein & Goodman, 2019).

Badenes et al. (2023) found that inappropriate practices also affected the willingness of yet-to-qualify health professionals to intervene in cases of domestic abuse. This reluctance was influenced by heightened sexist attitudes, which, in turn, led to greater acceptance of domestic abuse and a diminished sense of its severity. Prior research has shown that reluctance to intervene among social workers and law enforcement officers can also stem from patriarchal views, personal experiences with abuse, distorted portrayals of abuse in the media, survivor-blaming, and perceptions of the “typical” survivor (Murvartian, Saavedra-Macías, & de la Mata, 2023; Murvartian et al., 2024). Such attitudes and preconceptions can retraumatize victims and are at odds with the victim’s need for trauma-informed care, which is essential for building trust in patient–provider relationships (Battaglia et al., 2003). Frontline professionals, such as those in the police service, criminal justice system, social care, and healthcare (including nurses and midwives), play a vital role in preventing and managing domestic abuse (Hill, 2020; Jaffe et al., 2020; A. Millar et al., 2022). Recognizing this, the World Health Organization (2013) has published guidelines and recommendations for professionals to ensure their practice is trauma-informed.

Research indicates that first responders’ approaches to managing and preventing domestic abuse are strongly influenced by their professional knowledge, attitudes, preparedness, and training (Ali et al., 2022; Kirk & Bezzant, 2020; Munro & Aitken, 2020). Additionally, victims’ responses to clinical practices are largely dependent on the receptiveness of the professionals involved (Trevillion et al., 2012). Health, social care, and criminal justice professionals are most likely to receive disclosures of domestic abuse and are considered the frontline professionals in this study. Their intervention is crucial to managing and preventing domestic abuse, and it often significantly influences the healing process of the victims.

### The Current Review

At the time of this review, and to the best of our knowledge, only five systematic reviews have assessed public and professional attitudes, knowledge, or stigma surrounding domestic abuse. Kennedy and Prock (2018) examined female victims’ perceptions in the United States, revealing issues such as victim-blaming, denial, and discrimination by professionals. Gracia et al. (2020) reviewed general perceptions and attitudes toward domestic abuse in Europe and highlighted the need for more region-specific research.

Another U.S.-based review identified barriers to help-seeking among victims of intimate partner sexual violence, highlighting how stigma and rural isolation hinder support efforts (Wright et al., 2022). Serrano-Montilla et al. (2023) focused exclusively on police attitudes, finding that intervention was shaped by factors such as tolerance toward domestic abuse, understanding of the issue, and perceptions of professional responsibility. Finally, Murvartian, Saavedra-Macías, and Infanti (2023) global review examined stigma toward female victims of intimate partner violence in high-income countries, emphasizing both public and professional perceptions and their impact on victim outcomes. These reviews highlight that attitudes toward domestic abuse are variable and context-dependent, with negative attitudes and stigma having a profound impact on victims. However, a significant gap remains in documenting the current responses and attitudes of frontline professionals in Europe. Gracia et al. (2020) specifically called for more in-depth, region-specific research. This review addresses that gap by focusing on areas previously overlooked, such as the predominance of U.S.-based or global victim perspectives, the emphasis on public or general attitudes, the restriction to high-income countries, or the examination of a single professional group.

The present review focuses on current European studies, irrespective of country income status, and is conducted 10 years after the implementation of the Istanbul Convention. It examines a broad range of frontline professions and their responses to all forms of domestic abuse (financial, sexual, physical, psychological, and emotional abuse), and includes victims of all genders. The review also explores the mechanisms influencing frontline professionals’ responses, distinguishing between victim perceptions and professionals’ self-reported motivations. It aims to synthesize perceived inconsistencies in professional practice, as research has shown that victims’ recovery can be significantly influenced by professional attitudes (McLaughlin et al., 2024). The goal is to offer insights into how to better support both professionals and victims.

### Theoretical Framework

Various theories inform this review. Lipsky’s (2010) Street-Level Bureaucracy theory emphasizes that public service workers (such as social workers, police officers, teachers, and healthcare providers) exercise considerable discretion in performing their duties. According to Lipsky, the actions and decisions of these bureaucrats are not solely dictated by formal rules and policies but are also shaped by both proximal and distal environmental factors. These may include their personal backgrounds, judgments, resource limitations, and the broader constraints or realities of their work settings. In addition, Kahn et al.’s (1964) role stress theory proposes that individuals in certain roles, including street-level bureaucrats, may experience stress when there is a misalignment between the expectations placed upon them (from both internal and external sources) and the resources or capabilities they possess to meet those demands. Both theories support the notion that role-related stress can significantly influence professional practice. Furthermore, Schein’s (1983) model of organizational culture provides a foundation for understanding how organizational culture operates not merely at a surface level but is embedded within the underlying beliefs, values, and behaviors of the organization and its members. This theory underscores the importance of the organizational environment in shaping how street-level bureaucrats perform their duties and make decisions.

### Aims of Review

This review will:

(1) Identify frontline professional attitudes, responses, knowledge, and competencies regarding domestic abuse.
(2) Explore how psychosocial factors such as empathy and perceived sexism influence frontline professionals’ responses to domestic abuse.
(3) Identify the practical consequences of frontline professionals’ knowledge, attitudes, and competencies upon their professional practice within the context of trauma-informed care.

For this review, “frontline professionals” include doctors, nursing and midwifery staff, social care staff, police officers, and criminal justice practitioners (such as judges, solicitors, barristers, prosecutors, and magistrates).

## Method

### Study Design

This review is reported in accordance with the Preferred Reporting Items for Systematic Reviews and Meta-Analyses (PRISMA) guidelines (Kim & Royle, 2024), and the protocol is registered in PROSPERO (R. Millar et al., 2024). The strategy for data synthesis was guided by Thomas and Harden’s (2008) framework, specifically employing thematic synthesis, in which data were analyzed through the identification and integration of key themes. The umbrella terminology of “attitudes, knowledge, and competencies” used in this review was adapted from Tierney et al.’s (2022) protocol. Literature was sourced from the databases APA PsycInfo, Scopus, Web of Science, PubMed, Sociological Abstracts, International Bibliography of the Social Sciences, and Social Services Abstracts. Google Scholar was also consulted to identify additional published, non-duplicate literature.

### Inclusion Criteria

Women’s Aid’s (2024a) definition of domestic abuse was used to guide the search parameters: “an incident or pattern of incidents of controlling, coercive, threatening, degrading and violent behavior, including sexual violence, in the majority of cases by a partner or ex-partner, but also by a family member or carer.” The search included studies documenting professional responses to both adult victims (aged 18 and over) and child victims (under 18). Only domestic abuse victimization within Europe was considered. Victims and professional respondents of all ethnicities, sexual orientations, and genders were eligible for inclusion. To be included, studies were required to focus on first-hand accounts of professional attitudes, knowledge, and competencies related to domestic abuse.

Attitudes referred to the responses and assessments made by medical, legal, police, and social work professionals regarding domestic abuse survivors, as measured through quantitative tools, such as the Attitudes toward Domestic Violence Questionnaire (Fox & Gadd, 2012) or explored through qualitative methodologies. Knowledge encompassed both the actual and perceived understanding of domestic abuse among frontline professionals, with a particular focus on their self-assessed readiness and perceived ability to support victims. Competence was defined as the practical application of readiness, preparedness, and capability required for professionals to effectively fulfill their roles (Tierney et al., 2022). Studies based on domestic abuse victims’ perceptions of professional attitudes were excluded from this review. Eligible studies focused exclusively on frontline professionals, defined as medical and social care staff, police officers, and criminal justice practitioners. The review included studies published between 2014 and 2025 to ensure the inclusion of contemporary European research conducted within the 10 years following the implementation of the Istanbul Convention (Council of Europe, 2024).

Qualitative, quantitative, and mixed-methods studies were eligible for inclusion. Studies were excluded if they were not written in English, did not focus explicitly on domestic abuse, lacked full-text access, were systematic or scoping reviews (including reviews of interventions), or constituted unpublished grey literature. Grey literature refers to a broad range of documents produced by government agencies, academic institutions, businesses, and industries in both print and digital formats. While such materials are often protected by intellectual property rights and may meet quality standards suitable for inclusion in institutional repositories or libraries, they are not distributed by commercial publishers. In other words, the primary purpose of the producing entity is not publication (Schöpfel, 2010).

### Search Strategy

A comprehensive search and review of the literature was conducted across seven databases and Google Scholar, subsuming the fields of sociology, psychology, social work and care, medicine, and criminal justice. Three main search concepts were extracted: *domestic abuse* AND *professionals* AND *responses.* Related terms such as domestic violence, intimate partner violence, coercive control, and interpersonal violence were also used. Searches for the aspect of “professionals” were extrapolated into first responders, police, nurses, midwives, criminal justice, and social services. A full list of used search terms can be found in Table 1.

**Table 1.** Search Terms Used to Find Potentially Relevant Papers.

1	domestic abuse OR domestic violence OR intimate partner violence OR coercive control OR interpersonal violence
2	professional* OR first responder* OR police OR policing OR nurs* OR midwife* OR criminal* OR justice* OR judicia* OR “criminal justice” OR “legal processes” OR law enforcement OR police personnel OR adjudication OR protective services OR social services OR social work* OR community services
3	response* OR perception* OR attitude* OR knowledge OR competenc*

### Data Extraction and Analysis

The SPIDER (Sample, Phenomenon of interest, Design, Evaluation, and Research type) tool was used to extract and define aspects of the research question and inclusion criteria for the search strategy. Endnote and Rayyan were used to extract potentially relevant articles from the identified databases, which were reviewed independently by two researchers (R.M. and O.C.). Following PRISMA guidelines for screening and data checks, the relevant studies for inclusion were synthesized by extracting author(s), publication year, localization, participant population, methods and analysis, outcome, conclusions, and recommendations. Due to the heterogeneity of the studies’ samples and methodologies (qualitative, quantitative, and mixed-method) a narrative synthesis based on Popay et al.’s (2006) narrative guidance was employed.

## Results

### Study Selection

**Figure 1.**
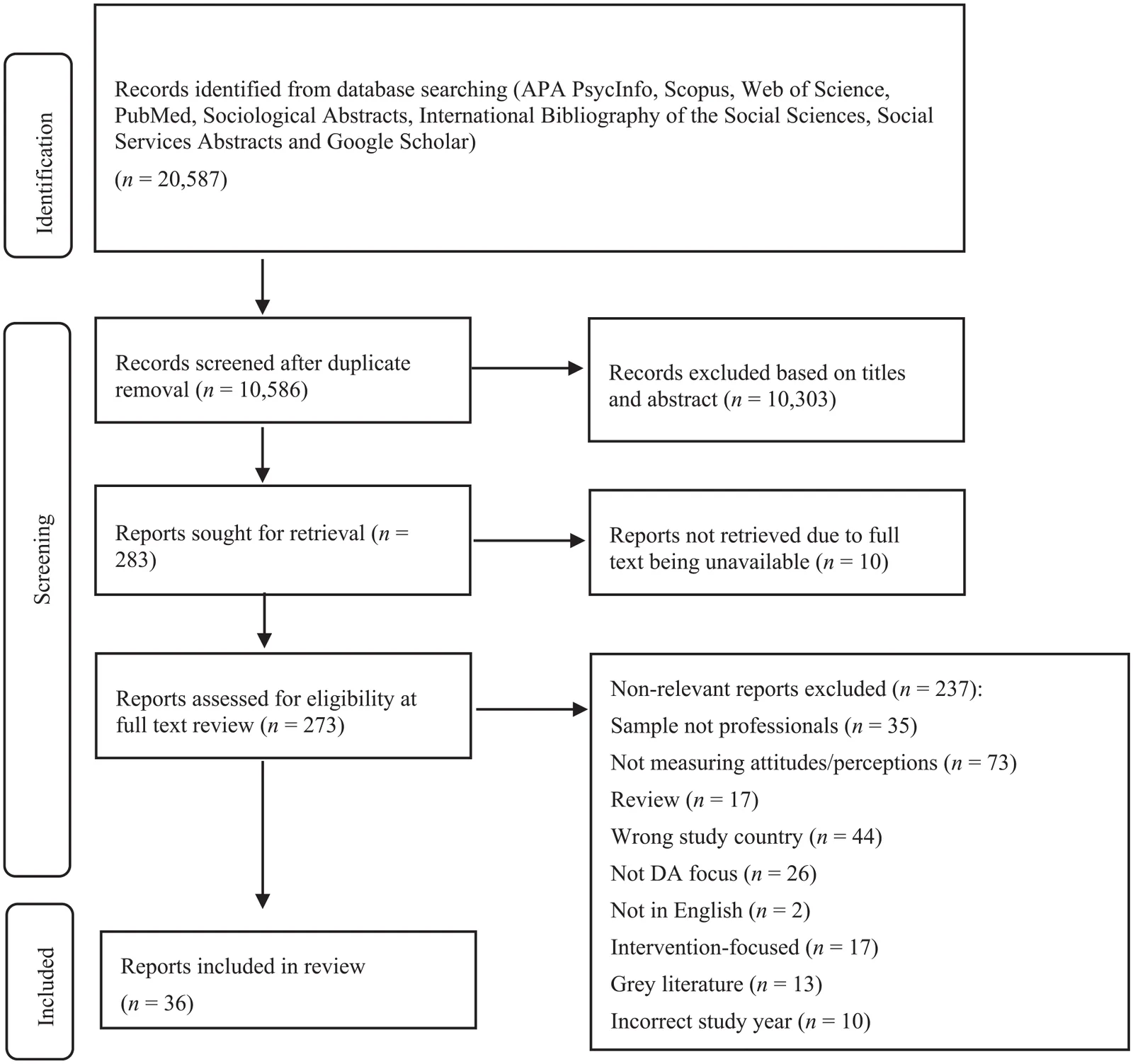
PRISMA flowchart of systematic review inclusion process (Moher et al., 2020). *Note*. PRISMA = Preferred Reporting Items for Systematic Reviews and Meta-Analyses.

Of the 20,587 records extracted from the relevant databases, 10,001 were duplicated and removed from the search. Subsequently, two authors (R.M. and O.C.) performed screening. The second reviewer was provided with the inclusion criteria and search terms and completed 25% of title/abstract and full-text screening. When screening varied between the reviewers, discussion took place based on study inclusion and exclusion criteria, until a consensus was reached regarding study inclusion or exclusion. Any discrepancies were discussed with the wider research team. 10,586 records were screened by title and abstract, resulting in the exclusion of 10,303 records. Retrieval of full-text was conducted on 283 records, with 10 unable to be retrieved due to not being open access. Full-text review was performed on the remaining 273 records, 237 were excluded as they: (a) did not sample relevant frontline professionals (*n* = 35), (b) did not measure attitudes/perceptions (*n* = 73), (c) were a review (*n* = 17), (d) were not conducted in Europe (*n* = 44), (e) were not focused on domestic abuse (DA) (*n* = 26), (f) were not in English (*n* = 2), (g) were intervention-focused (*n* = 17), (h) were grey literature not previously identified (*n* = 13), or (i) data collection was conducted prior to 2014 (*n* = 10). Finally, 36 records met the full inclusion criteria and were deemed appropriate to be included in the systematic review. Figure 1 shows a breakdown of the inclusion process in a PRISMA flowchart (Moher et al., 2020).

### Study Characteristics

The 36 articles included in the review were published between 2014 and 2024. The number of publications per year has remained relatively steady (*M* = 3), with the least publications occurring in 2020 and 2022 (*n* = 1 per year). The review included eight (22.2%) multi-country studies spanning the United Kingdom (England, Scotland, and Wales) and 28 (77.8%) single-country studies. Six were conducted in England (16.7%), six in Sweden (16.7%), five in Turkey (13.9%), two in Portugal (5.6%), three in the Republic of Ireland (1.3%), and one publication each from Wales, Bosnia, Spain, Italy, Slovenia, and Hungary (*n* = 1, 0.4%).

Sampling type was predominantly purposive sampling (*n* = 16, 44.4%). Six studies used convenience sampling (16.7%), four a combination of snowball and purposive sampling (11.1%), two snowball sampling alone (5.6%), and one random sampling (2.8%). Sampling methods for nine studies were unreported (*n* = 9, 25%). Study characteristics included qualitative, quantitative, and mixed-methods, and thus sample sizes varied. Of the included studies and from the available sample sizes, females were sampled significantly more frequently than males (31,854 and 1,472, respectively) across a variety of professions (nurses, midwives, social workers). Males formed most police officer samples, and criminal justice staff genders were evenly distributed.

Quantitative studies, or studies with a quantitative aspect, predominantly used surveys and questionnaires to assess attitudes and responses by frontline professionals toward domestic abuse. Samples ranged from 121 (Downie et al., 2019) to 787 participants (Lundberg & Bergmark, 2021). Qualitative studies, or those including qualitative aspects, utilized interviews or focus groups and ranged from samples of six (Naughton et al., 2015) to 61 participants (Robinson, Myhill, et al., 2018). One study conducted a synthesized analysis of 511 police records and 432 field observations (Myhill & Johnson, 2016), another performed a qualitative analysis of 350 hr of police field observations (Myhill et al., 2023). Most studies were solely conducted with police officers (*n* = 10, 27.8%), nurses and midwives (*n* = 6, 16.7%), social workers (*n* = 3, 8.3%), orthopedic staff (*n* = 1, 2.8%), doctors (*n* = 1, 2.8%), or judges (*n* = 1, 2.8%). The remaining studies used mixed sampling with various professions (*n* = 17, 47.2%). Table 2 shows a summary of study characteristics.

**Table 2.** Summary of Study Characteristics.

Author(s) Publication Year	Location	Design	Sample (*N*)	Profession(s) of Sample	Summary of Findings	Quality Appraisal
Ali et al. (2023)	United Kingdom	Quantitative convenience	220 females 18 males	Nurses and midwives	Participants had minimal previous DVA (Domestic Violence and Abuse) training and minimal knowledge about DVA. Although they had positive attitudes toward engaging with women experiencing DVA.	High^b^
Ali et al. (2022)	England and Wales	Quantitative convenience	739 females 30 males	Nurses and midwives	Participants find it challenging to manage cases of IPV (Intimate Partner Violence) due to limited knowledge and preparedness. Nurses and midwives also used the learning from past experiences of managing and experiencing IPV to manage new cases. Participants reported the role of personal and professional experiences of IPV promoted them to manage cases effectively.	High^b^
Anderzén Carlsson et al. (2021)	Sweden	Qualitative purposive	9 females	Children’s health care nurses	Knowledge and experience with DV (Domestic Violence) make nurses more confident in their interactions with patients. Nurses may not take immediate actions or initiate collaboration (with social services) if DV is suspected. There is often disappointment with available resources and outcomes for victims.	High^a^
Aregger Lundh et al. (2023)	Sweden	Qualitative purposive	2 males 7 females	Emergency department nurses	Short encounters in emergency care don’t allow nurses to have deep conversations. There is a need for more knowledge and how to interact with patients when DV is suspected. Some nurses avoid asking about DV due to lack of time/awkwardness in handling the answer.	High^a^
Çalıkoglu et al. (2018)	Turkey	Quantitative purposive	370 participants	Healthcare professionals	Less than half of participants felt competent in recognizing and managing VAW (Violence Against Women), with some indicating they would try to reconcile victims with perpetrators. Male participants scored higher on both VAW attitudes and sexist attitudes. Age and sexist attitudes were significant independent predictors of VAW attitudes. Recent exposure to VAW was associated with lower sexist and VAW attitude scores. Medical doctors showed the least biased scores.	High^b^
Clarke and Wydall (2015)	Wales	Qualitative	54, genders unknown	Health and social care, police, housing and third sector organizations	There is variation in children’s experiences of DA versus Parents. Participants are aware that DA can lead to a loss of childhood, with children not wanting to burden their mother for fear of guilt. Practitioners are very aware of the needs of children and respond empathetically to them.	High^a^
Donnelly and Holt (2021)	Republic of Ireland	Qualitative purposive	27, genders unknown	Social work, psychologists, occupational therapy, nurses, junior doctors	Routine screening for DA is often influenced by ambivalence based on victim attitudes. Healthcare professionals may not believe it’s their responsibility to track DA disclosures, viewing it as the victim’s responsibility and social worker’s responsibility. Lack of understanding of DA, with a focus on the biomedical model and absence of system support. Lack of knowledge on CC (Coercive Control) and the absence of physical evidence raised doubts about whether it’s DA. Professional avoidance due to fear, discomfort, and lack of awareness. Reduced hesitancy when it comes to physical violence, but medical bias and mental health diagnoses can lead to the belief that victims are lying. Cautiousness and disbelief result in the victim not being viewed as reliable.	High^a^
Downie et al. (2019)	United Kingdom	Quantitative Purposive	121, genders unknown	Orthopedic staff	52% of respondents had previously had a disclosure of IPV from at least one orthopedic patient. Doctors and nurses were equally likely to have cared for IPV patients, but doctors thought abuse was less common. 74% of respondents reported asking patients about abuse but only 24% knew about the support currently available. Staff who did not know about available support were less likely to ask about possible abuse.	High^b^
Eliasson and Morabito (2024)	Sweden	Qualitative snowball	27, genders unknown	Police officers	Police officers assess victims’ culpability based on their choices and ability to prevent victimization. Officers are more likely to show empathy toward victims who are seen as less responsible or unable to protect themselves, such as children or the elderly. Victims who make choices that increase their risk of victimization are perceived as less deserving of empathy. Officers also factor in a victim’s history, like past criminal behavior or prior experiences with DV, when evaluating culpability.	High^a^
Elliffe and Holt (2019)	Republic of Ireland	Qualitative purposive and snowball	10 females, 4 males	Police officers	Police officers don’t feel it’s their responsibility to respond to children as victims if they’re not direct victims. Some agreed their role was extended beyond the original victim but were unsure how to handle those situations and identify non-physical violence. Police require physically viewing the child to acknowledge them as a victim and needing support. Non-physical cues often lead to a lack of emotional support. Participants spoke of children’s perceived immunity to violence, stating they’re used to it and indicating a perceived lack of severity.	High^a^
Franco and Augusto (2024)	Portugal	Qualitative purposive	31, genders unknown	Nurses, doctors, social workers	Health professionals lack training, protocols, and confidence to respond to DV effectively. They often take a medical-focused approach instead of a comprehensive intervention. They struggle with screening, reporting, and addressing DV due to limited knowledge and resources. There are concerns about negative consequences of reporting and emotional burden. There is fear of breaching confidentiality and lack of understanding of CC. They acknowledge power imbalances between victims and their partners as barriers to disclosure.	High^a^
Gölge et al. (2016)	Turkey	Quantitative	85 females, 365 males	Police officers, judiciary members	Police officers are more tolerant of physical and verbal abuse in marriages, especially regarding a victim leaving an abusive partner. Male officers are more tolerant of abusive behavior and less supportive of a wife leaving her abusive husband compared to female officers. Police officers held more negative attitudes toward wife abuse than judiciary members. Profession had a stronger influence on attitudes toward wife abuse than gender. Ambivalent sexism was a key predictor of these attitudes. Both male and female police officers had more negative views on wife abuse than judiciary professionals.	High^b^
Gracia et al. (2014)	Spain	Quantitative purposive	308 males	Police officers	Results showed that police officers prefer a stronger and unconditional law enforcement approach in cases of violence against women, both in intimate and non-intimate relationships. Benevolent sexism was linked to a preference for a more conditional law enforcement across interpersonal violence scenarios. The type of interpersonal violence scenario also conditioned the influence of hostile sexism and empathy on police preferences.	High^b^
Heffernan et al. (2014)	England	Mixed-method purposive	19 qual females, 149 quant females, 30 quant males	Social workers	Participants recognize physical and non-physical abuse. Male social workers are more likely to believe 1/4 people experience abuse. Men were more likely to say DA is caused by drugs/alcohol and more likely to think witnessing abuse as a child causes DA in adulthood. Participants agreed women are entitled to help and there needs to be more funding. Victim blaming about women not leaving was present and participants identified the need for more training and awareness to DA policies.	High^a^
Lundberg and Bergmark (2021)	Sweden	Quantitative, random	787, genders unknown	Social workers	IPV is a widespread issue among Swedish social services clients, but many social workers feel unprepared to handle it. Training, self-perceived competence, and administrative procedures positively influenced social workers’ likelihood of addressing IPV. Two-thirds of respondents had received IPV training in the past 3 years, with variation based on specialization. Social workers with training reported higher competence and were more likely to inquire about IPV.	High^b^
Martins et al. (2023)	Portugal	Quantitative convenience	253 females, 26 males	Doctors, nurses, psychologists, and social assistants	Most healthcare professionals do not follow current guidelines for screening and intervening in IPV during pregnancy and postpartum (IPV-PPP). Barriers include poor communication skills and lack of IPV training. Psychologists were more likely to engage in screening and intervention than other professionals. Positive correlations were found between screening and intervention practices. Modifiable barriers identified include healthcare professionals’ individual characteristics, knowledge, and attitudes.	High^b^
Muftić and Cruze (2014)	Bosnia	Quantitative convenience	10 females, 127 males	Police officers	Officers who received DV training were less likely to hold sexist views and more likely to recognize DV as requiring police action. Overall, few differences were found between officers who had received training and those who had not. Officers with conservative or traditional views on women were more likely to justify wife beating. Officers with negative views of women or who justified wife beating were less likely to support police intervention in DV cases. Bosnian officers who received DV training had slightly lower scores on the ATW (Attitudes Toward Women) measure compared to those who did not.	High^b^
Munro et al. (2024)	United Kingdom	Qualitative purposive and snowball	45, genders unknown	Police officers, Crown Office prosecutors, sheriffs, magistrates, and third-sector specialists	Participants are aware that some female offenders will have been victims of DA but they lack the clarity toward the impact this has on their offending. Participants are unclear on how to prosecute CC and exhibit attitudes of victim blaming, “why didn’t she leave?” They also indicated a lack of ambition to recognize CC and believe it is the jury’s responsibility to identify it. Overall, they displayed attitudes of diminished responsibility, less sympathy for CC and a tendency to diminish the impact of abuse.	High^a^
Myhill and Johnson (2016)	England	Mixed-method purposive	943 quant cases, 5 qual teams	Police officers	Police officers often use discretion when categorizing “disputes” not as DA. Participants stated that DA doesn’t get recorded as DA when others believe it should. There is a perceived lack of severity toward certain aspects of abuse, such as non-physical abuse, which contributes to the trivialization of abuse. DA was often diminished in the fact of other “more serious” offences.	High^a^
Myhill et al. (2023)	England	Qualitative	350 hr police field observations	Police officers	The study found that the CC law is not effectively implemented, with police struggling to identify and prosecute such cases. Challenges include a high evidential threshold, normalized controlling behaviors, and gendered assumptions. Limited resources lead to a reactive approach, prioritizing high-risk cases and physical assaults over CC, which is seen as difficult to prove.	High^a^
Naughton et al. (2015)	Republic of Ireland	Qualitative purposive and snowball	4 females, 2 males	Judges	Judges recognized the impact of DA but believed that having the father in the child’s life was more important than the presence of DA. There was a consistent lack of severity toward CC and a belief that some mothers are problematic victims. Some judges labeled mothers who wanted to restrict contact as manipulative and self-serving, viewing their claims of DA as a way to gain the upper hand in custody proceedings. They also used previous abusive relationships to blame the victim and believe that it’s the responsibility of the victim to protect the children. One judge only promoted contact if they believed it was valuable to the child.	High^a^
Öhman et al. (2024)	Sweden	Qualitative	15 females, 3 males	Doctors, nurses, midwives, psychologists, and physiotherapists	Significant variation in primary care professionals’ awareness and understanding of VAW. Some professionals are highly knowledgeable, while others are less so. Many professionals struggle with asking patients about violence and suggested improvements at the professional, managerial, and organizational levels. They can identify signs of violence, such as bruises and scars, but often lack the skills and confidence to address it directly.	High^a^
Özdemir et al. (2023)	Turkey	Quantitative	294, genders unknown	Doctors, nurses, and midwives	Health professionals had more negative attitudes toward DV but more positive attitudes toward reporting violence against women. Ethical dilemmas related to privacy and confidentiality were common. Health professionals who believed they were better equipped to handle violence were more likely to report it. Health professionals’ responsibilities in DV cases were unclear, highlighting the need for clear guidelines and action models for intervention.	High^b^
Parti and Robinson (2021)	Hungary	Qualitative purposive	22, genders unknown	Police officers, prosecutors, and judges	Hungary’s criminal justice system does not prioritize victims’ needs, focusing mainly on legal justice. There was a lack of standardized protocols for addressing victims’ needs, insufficient technical knowledge and training, and a shortage of trauma-informed services for practitioners. Underreporting of sexual violence linked to procedural complexities, inadequate education and training, and cultural attitudes.	High^a^
Pitt et al. (2020)	England and Wales	Qualitative convenience	17 females, 13 males	General practitioners, police and partnership managers	GPs (General Practitioners) think knowing about DA is useful. They know of the adverse health impacts of DA and what GPs contribute. They are supportive of information sharing but there are dilemmas about how to record DA in health contexts—how to redact, what is confidential, fears about assigning labels. Aware of the vulnerability factors, concerns about causing an escalation,	High^a^
Procentese et al. (2019)	Italy	Qualitative purposive	24 females, 6 males	Obstetricians, nurses, trainees, medical students, a health practitioner, gynecologists, and first aid police officers	Healthcare professionals often do not view DV as a direct concern for their health services and lack adequate training and support to manage cases effectively. Obstetricians and other health personnel expressed negative attitudes toward asking about violence and abuse in prenatal reports. Health professionals recognize DV as a critical issue and desire to create supportive environments for victims.	High^a^
Richards et al. (2021)	England	Qualitative purposive & snowball	12, genders unknown	Police officers	Participants have a desire to help but lack training. Inconsistencies in support lead to victim blaming and frustration. There is a lack of understanding toward DA, with participants struggling to relate to the victim. They expressed anger toward the perpetrator. There is also a perceived lack of severity to non-physical abuse and a tendency to view victims by typology. Participants also demonstrated an awkwardness with handling DA and desensitization when responding to victims.	High^a^
Robinson, Pinchevsky, et al. (2018)	United Kingdom	Mixed-method purposive	5 females, 260 males	Police officers	Police officers show a lack of understanding to CC, diminishing its severity. They also expressed frustration to being called out for “minor issues,” showing frustration toward victims. There was a lack of understanding of how to record DA and officers would not record risk assessments for “verbal only” altercations. While some officers understood CC, they still could not understand how the perpetrator may control the victims’ behavior e.g. views of recording being a “waste of time” for something that wasn’t reported.	High^a^
Robinson, Myhill, et al. (2018)	England and Wales	Mixed-method	61 qual, 1419 quant	Police, partner agencies and Independent Domestic Violence Advisors	Findings indicated that British and American ofﬁcers were largely in agreement about a small constellation of risk factors that they considered integral to the risk assessment process: using or threatening to use a weapon; strangulation; physical assault resulting in injury and escalation of abuse. The results revealed that ofﬁcers’ country of employment, rather than their demographic characteristics or experience policing DA, was a particularly inﬂuential predictor of their perceptions, and that both the situational context and the victim’s perception about risk are important in DA risk assessment.	High^a^
Robinson et al. (2016)	United Kingdom	Mixed-method purposive	5 females, 260 males	Police officers	The findings showed that most officers in both countries had a solid understanding of DA and how to respond to it, including beyond just physical violence. However, physical violence tends to be the primary focus for many officers when it comes to DA, and without physical violence, their response tends to be less proactive. Non-physical abuse often goes unnoticed by some officers, with this being more common among American officers compared to their British counterparts.	High^^a^^
Simsek-Cetinkaya and Evrenol Ocal (2023)	Turkey	Qualitative snowball	17, genders unknown	Nurses and midwives	Midwives and nurses face individual, institutional, and political challenges when addressing DV against women. They feel inadequate and unprepared to recognize DV symptoms and support affected women. Key themes identified: Difficulty recognizing violence; Obstacles to revealing DV; Challenges in supporting pregnant women who are DV victims; Potential solutions to prevent violence in pregnancy; Institutional deficiencies in identifying and intervening in DV cases.	High^a^
Sundborg et al. (2017)	Sweden	Qualitative purposive	9 females	Nurses	The study identified the “hesitation process” as a central concept in DNs (District Nurses) encounters with women exposed to IPV. DNs’ beliefs and attitudes toward asking about IPV are influenced by factors such as cultural differences, age-related perspectives, and a lack of strategies for asking. DNs’ hesitation was influenced by fear, shame, guilt, and the availability of training, strategies, and professional support.	High^a^
Taskiran et al. (2019)	Turkey	Quantitative purposive	266, genders unknown	Primary healthcare workers (PCW)	52.6% of PCWs responded appropriately to IPV disclosures, with family physicians more likely to take appropriate action than midwives/nurses. Key issues: high rate of IPV disclosures but low rate of appropriate responses. Barriers to effective management include physician-level challenges, patriarchal attitudes, and an inefficient justice system response. Identified lack of knowledge and training in managing IPV cases, as well as a lack of specialized support systems for PCWs. Justice system inefficiencies, such as police reluctance to arrest perpetrators and lack of empathy from justice personnel, were significant barriers to proper IPV management.	High^b^
Wiener (2017)	England	Qualitative	20, genders unknown	Police officers and Independent Domestic Violence Advisors	There is a perceived lack of severity toward DA, with professionals not counting CC as DA. They often initially assess CC as low risk and look at incidents of abuse in an isolated manner. There is a consistent lack of understanding of CC and participants reported a lack of training when taking statements.	Medium^a^
Witt and Diaz (2019)	England	Qualitative purposive	9, genders unknown	Social workers	Social workers recognize the challenges DV presents for abused mothers in safely parenting their children. DV is acknowledged as a significant issue in child protection, mainly due to its harmful effects on child development. Social workers expressed a need for further training on DV. Emphasized the importance of supporting mothers for positive child outcomes, but also noted contradictions in their work where interventions may not always feel supportive.	High^a^
Zorjan et al. (2017)	Slovenia	Quantitative convenience	274 females, 48 males	Doctors, dentists, healthcare professionals, social workers, administrative staff and psychologists	No significant differences in DV acceptability attitudes between healthcare professionals who reported encountering low or high frequencies of DV cases. Negative association found between the acceptability of DV attitudes and action-taking, especially when the frequency of encounters was high or medium. Attitudes toward the acceptability of DV were statistically correlated with action-taking, but not with the frequency of encounters.	Medium^b^

*Note*. Qual = Qualitative; Quant = Quantitative. High = >70%; Medium = 50% to 70%. DA = domestic abuse; DV = domestic violence; IPV = intimate partner violence; VAW = violence against women; ATW = attitudes toward women; CC = coercive control; DN = district nurse; PCW = primary care worker; GP = General Practitioner.

a CASP UK Checklist (out of 10 [100%]).

b JBI Checklist (out of 8 [100%]).

### Quality Assessment

Two researchers (R.M. and O.C.) performed screening and quality assessments, evaluating biases in the studies identified for inclusion. Bias assessments were conducted in line with the Critical Appraisal Skills Program (CASP) and the Joanna Briggs Institute (JBI) critical appraisal checklists (CASP UK, 2024; JBI, 2024). Checklists were alternated for appropriateness based on study type (qualitative, quantitative, mixed-methods). Scores were generated based on George et al.’s (2014) quality assessment criteria: high scores were over 70% and judged to be excellent quality, medium were between 50% and 70% (satisfactory quality), and low-quality articles were less than 50% (of which there were none). Percentages were out of 10 (100%) (CASP UK, 2024) and 8 (100%) (JBI, 2024). Table 2 summarizes study quality scores.

### Categories and Measures

By consensus of the reviewers, codes were manually derived through a narrative synthesis. Given their conformity and similarities to Gracia et al.’s (2020) and Villagrán et al.’s (2024) multidimensional categorization, they were incorporated into four categories: responsibility (*n* = 22, 61.1%) permissiveness (*n* = 20, 55.6%) attitudes toward domestic abuse (*n* = 28, 77.8%) and practice (*n* = 23, 63.9%). Responsibility included codes like training, duties, obligations, and accountability. Permissiveness included codes like victim blaming, minimization, and legitimization. Attitudes toward domestic abuse included codes like knowledge, perceptions, and perceived severity. Finally, practice included codes like routinization and normalization.

Most studies were qualitative (*n* = 19, 52.8%) or quantitative (*n* = 12, 33.3%), with five being mixed-method (13.9%). Of the quantitative scales used (*n* = 15), six used validated attitudinal measures (40%) based on existing scales (Ali et al., 2022, 2023; Downie et al., 2019; Gölge et al., 2016; Gracia et al., 2014; Özdemir et al., 2023) and nine used ad hoc scales (Çalıkoglu et al., 2018; Heffernan et al., 2014; Lundberg & Bergmark, 2021; Martins et al., 2023; Muftić & Cruze, 2014; Robinson et al., 2016; Robinson, Pinchevsky, et al., 2018; Taskiran et al., 2019; Zorjan et al., 2017). Of those availing of ad hoc scales, four investigated and reported the reliability and validity of the instruments (Çalıkoglu et al., 2018; Heffernan et al., 2014; Lundberg & Bergmark, 2021; Martins et al., 2023) while the remaining five did not report reliability nor validity.

### Themes

While initially guided by the reviews of Gracia et al. (2020) and Villagrán et al. (2024), the current study’s novel findings led to the derivation of new themes. Popay et al.’s (2006) narrative synthesis and Thomas and Harden’s (2008) thematic synthesis frameworks were consulted during the theme development process. The primary researcher (R.M.) undertook the following steps: (a) developed a theory on how attitudes and perceptions are shaped by psychosocial factors and various theoretical frameworks, including Street-Level Bureaucracy (Lipsky, 2010), Role Stress Theory (Kahn et al., 1964), and the Model of Organizational Culture (Schein, 1983); (b) conducted a preliminary synthesis of the included studies through coding; (c) examined the relationships between the codes and data; (d) assessed the robustness of the synthesis; and (e) defined the final theme names.

The themes constructed were: attitudes toward engagement and responsibility, attitudes toward victims, knowledge and understanding of domestic abuse, and attitudes as predictors of professional practice. Given the iterative nature of the synthesis process and the frequent variability of attitudes, significant overlap was observed within and between studies. This overlap is highlighted throughout the results section.

### Attitudes Toward Engagement and Responsibility

This theme encompassed 23 studies (63.9%) that documented frontline professionals’ attitudes, knowledge, and competencies regarding their perceived responsibilities and engagement with domestic abuse legislation, victims, and training. Attitudes toward engagement and responsibility were consistent across all nations included in the studies. The articles represented countries such as Sweden, Turkey, Portugal, Bosnia, Italy, Great Britain, and the Republic of Ireland. Within this theme, multiple attitudes toward engagement and responsibilities were identified. Of the studies, 16 depicted perceived competencies, 14 highlighted disengagement, and 9 demonstrated enthusiasm for addressing domestic abuse.

Police officers and criminal justice staff often disengaged from domestic abuse cases, frequently neglecting their responsibilities (Myhill et al., 2023; Parti & Robinson, 2021). They were more likely to perceive domestic abuse as a non-criminal matter and tended to shift the responsibility for prosecution onto the victim (Richards et al., 2021; Robinson, Pinchevsky, et al., 2018), while delegating victim support to other professionals (Munro et al., 2024; Richards et al., 2021). Similar disengagement was noted among healthcare professionals, including general practitioners, nurses, and social workers, who often did not perceive it as their responsibility to address domestic abuse (Aregger Lundh et al., 2023; Donnelly & Holt, 2021; Procentese et al., 2019; Sundborg et al., 2017; Witt & Diaz, 2019).

However, these attitudes may stem from frustration, anger, and uncertainty, with many frontline professionals citing incompetency, lack of training, time constraints, and heavy workloads as reasons for disengagement (Aregger Lundh et al., 2023; Çalıkoglu et al., 2018; Donnelly & Holt, 2021; Richards et al., 2021). Despite these challenges, many professionals also expressed positive attitudes toward supporting victims and enthusiasm for education and intervention (Ali et al., 2023; Anderzén Carlsson et al., 2021; Clarke & Wydall, 2015; Donnelly & Holt, 2021; Elliffe & Holt, 2019; Franco & Augusto, 2024; Öhman et al., 2024; Pitt et al., 2020; Simsek-Cetinkaya & Evrenol Ocal, 2023). Some police officers saw their role as extending beyond prosecution, in contrast to others who minimized their responsibilities (Elliffe & Holt, 2019; Muftić & Cruze, 2014; Myhill et al., 2023; Richards et al., 2021). Social workers and medical professionals were generally more enthusiastic about supporting victims (Ali et al., 2023; Anderzén Carlsson et al., 2021; Clarke & Wydall, 2015; Donnelly & Holt, 2021; Franco & Augusto, 2024; Öhman et al., 2024; Pitt et al., 2020; Simsek-Cetinkaya & Evrenol Ocal, 2023).

### Attitudes Toward Victims

This category included 20 articles (55.6%) depicting professionals’ perceptions of victims and victimization. Attitudes toward victims were frequently crosscutting between articles, with 13 articles reflecting attitudes of empathy and understanding, 10 focusing on minimization and dismissal of experiences, and 13 highlighting victim-blaming. These articles represented studies from Turkey, Slovenia, Sweden, England, and Bosnia. As with other themes in this review, many articles presented more than one attitude, with frequent overlap.

Attitudes of dismissal, minimization, victim-blaming, and lack of empathy were common among police officers and criminal justice professionals (Eliasson & Morabito, 2024; Elliffe & Holt, 2019; Gölge et al., 2016; Muftić & Cruze, 2014; Munro et al., 2024; Myhill et al., 2023; Naughton et al., 2015; Richards et al., 2021; Robinson, Myhill, et al., 2018; Robinson, Pinchevsky, et al., 2018). Police officers were found to dismiss domestic abuse callouts as “minor issues” and often lacked empathy (Robinson, Pinchevsky, et al., 2018). Despite recognizing the complexities of domestic abuse, they tended to minimize its impact (Richards et al., 2021). These attitudes led to victim-blaming, with officers questioning why victims didn’t leave or accusing repeat victims of “crying wolf” (Eliasson & Morabito, 2024; Munro et al., 2024; Richards et al., 2021). Richards et al. (2021) identified that frontline professionals categorized victims based on perceived “believability,” such as the “Genuine Victim,” or “Manipulative Victim.”

Negative attitudes were also present among some health and social care professionals (Çalıkoglu et al., 2018; Clarke & Wydall, 2015; Donnelly & Holt, 2021; Heffernan et al., 2014; Özdemir et al., 2023; Taskiran et al., 2019; Witt & Diaz, 2019), with some questioning victims’ experiences when physical evidence was lacking (Donnelly & Holt, 2021) or blaming victimized parents for inadequate child protection (Clarke & Wydall, 2015). However, attitudes varied, with some professionals recognizing the complexities of abusive relationships (Clarke & Wydall, 2015; Heffernan et al., 2014; Zorjan et al., 2017). Nurses, midwives, and general practitioners were generally more empathetic and aware of factors that might prevent victims from disclosing abuse (Ali et al., 2023; Pitt et al., 2020; Zorjan et al., 2017). Yet, some medical staff minimized victims’ experiences, particularly when co-morbidities like mental health issues or disabilities were present, labeling victims as “unreliable witnesses” (Donnelly & Holt, 2021). This suggests that vulnerable victims may face additional barriers in being believed and supported.

### Knowledge and Understanding of Domestic Abuse

Most articles included in this review demonstrated professionals’ knowledge and understanding of domestic abuse (*n* = 24, 66.7%). However, expressions of knowledge and understanding were often contradictory and crosscutting between articles. Fifteen articles included frontline professionals’ perceptions of a severity scale for domestic abuse, 12 displayed informed or misinformed perceptions, and 6 articles noted the routinization of domestic abuse. Most articles within this category focused on professionals in Great Britain and the Republic of Ireland (*n* = 16), with three in Sweden, and one each in Spain, Turkey, and Bosnia.

The categorization of domestic abuse incidents was often based on a perceived severity scale, with misinformation and routinization of domestic abuse incidents present across all professions sampled. While professionals acknowledged coercive control and non-physical abuse, they frequently categorized abuse severity, with physical abuse considered the most severe (Anderzén Carlsson et al., 2021; Munro et al., 2024; Robinson et al., 2016; Wiener, 2017). For example, police officers tended to show more empathy for vulnerable victims, such as children or the elderly (Eliasson & Morabito, 2024), but this was often coupled with minimizing the impact of non-physical abuse and a lack of understanding of coercive control (Donnelly & Holt, 2021; Heffernan et al., 2014; Myhill & Johnson, 2016; Myhill et al., 2023; Robinson, Myhill, et al., 2018). This trend was most common among police and criminal justice professionals but was also observed in healthcare and social care staff (Anderzén Carlsson et al., 2021; Aregger Lundh et al., 2023; Özdemir et al., 2023).

In contrast, some medical and social care professionals recognized coercive control as a major risk factor for intimate partner homicide and the escalation of abuse (Clarke & Wydall, 2015; Downie et al., 2019; Robinson, Pinchevsky, et al., 2018), though some failed to recognize the subtle signs of domestic abuse (Aregger Lundh et al., 2023). Interestingly, a Spanish study showed that some police officers were aware of the severity of coercive control (Gracia et al., 2014). Medical and social care professionals were also generally more attuned to the challenges victims face in disclosing abuse (Clarke & Wydall, 2015; Pitt et al., 2020).

These factors contributed to the routinization and normalization of domestic abuse in practice. Police, criminal justice staff, and social workers often underestimated abuse rates and effects (Elliffe & Holt, 2019; Heffernan et al., 2014; Lundberg & Bergmark, 2021; Muftić & Cruze, 2014; Richards et al., 2021). Some officers referred to domestic abuse as their “bread and butter” and only took notice of exceptional cases (Richards et al., 2021). Social workers in Sweden reported not asking about domestic abuse unless there was a “special reason” (Lundberg & Bergmark, 2021), while officers in Ireland often dismissed the effects of abuse on children unless the child was visibly upset (Elliffe & Holt, 2019). Judges in Ireland also prioritized maintaining “nuclear family” structures, often minimizing coercive control and viewing it as less serious compared to other forms of abuse (Naughton et al., 2015).

### Attitudes as Predictors of Practice

Each of the attitudes, knowledge and competencies identified within the preceding themes were significant predictors of practice within all frontline professionals sampled, and were representative of the first aim of this review. Twenty-six articles explored the third aim of the study, identifying the practical consequences of professionals’ knowledge, attitudes, and competencies. Professional practice can be categorized into two types: reactive and proactive. The studies included in this section were representative of Great Britain, Spain, the Republic of Ireland, Turkey, Hungary, Slovenia, Portugal, and Bosnia.

Proactive intervention in domestic abuse cases was linked to professionals’ ability to empathize, relate, and recognize the need for interventions. Around 25% of articles (*n* = 9) demonstrated professionals taking proactive action, particularly those with prior experience or specialized domestic abuse training (Ali et al., 2022; Downie et al., 2019; Gracia et al., 2014; Lundberg & Bergmark, 2021; Muftić & Cruz, 2014; Pitt et al., 2020; Robinson, Pinchevsky, et al., 2018; Sundborg et al., 2017; Witt & Diaz, 2019). Training enhanced empathy, proactive intervention, and recognition of non-physical abuse as a risk factor for homicide (Downie et al., 2019; Lundberg & Bergmark, 2021; Muftić & Cruz, 2014; Robinson, Pinchevsky, et al., 2018; Sundborg et al., 2017). In contrast, reactive practices, found in 47.2% of articles (*n* = 17), involved minimizing or disregarding victims’ experiences, leading to callousness, disengagement, desensitization, and victim blaming (Ali et al., 2023; Çalıkoglu et al., 2018; Clarke & Wydall, 2015; Donnelly & Holt, 2021; Elliffe & Holt, 2019; Gölge et al., 2016; Martins et al., 2023; Munro et al., 2024; Myhill & Johnson, 2016; Naughton et al., 2015; Özdemir et al., 2023; Parti & Robinson, 2021; Richards et al., 2021; Robinson et al., 2016; Robinson, Myhill, et al., 2018; Wiener, 2017; Zorjan et al., 2017). These attitudes were often influenced by a lack of training, insufficient professional practice knowledge, frustration, and heavy workloads (Martins et al., 2023; Parti & Robinson, 2021).

Exploring the second aim of this study, psychosocial variables like gender, sexism, empathy, and conservative beliefs impacted professional practices. A Spanish study (Gracia et al., 2014) found that male police officers with fewer sexist beliefs were more likely to enforce laws strictly and provide more victim support. Empathy was also a key predictor of proactive intervention. Additionally, male professionals, particularly in healthcare and law enforcement, were more likely to exhibit negative attitudes, including victim blaming, compared to female professionals (Çalıkoglu et al., 2018; Gölge et al., 2016; Özdemir et al., 2023). Culture also played a role, with Bosnian police officers holding liberal, pro-feminist attitudes, while conservative beliefs were associated with the justification of “wife beating” (Muftić & Cruze, 2014). These psychosocial factors reflect broader social, organizational, and cultural influences, rather than being profession-specific, and contribute to the complexities of professional responses to domestic abuse.

## Discussion

This review is the first, to our knowledge, to focus on professional responses to all forms of domestic abuse across European countries, regardless of income level, and to consider victims of any gender. It also examines the psychosocial factors shaping these responses. Our findings reveal a significant lack of domestic abuse-specific training for front-line professionals and criminal justice practitioners, which leads to negative attitudes with serious consequences for both professionals and victims (Gracia et al., 2020; Serrano-Montilla et al., 2023; Villagrán et al., 2024). This review spans a range of professions and offers crucial insights into improving professional practices and policies. Based on the frameworks of Serrano-Montilla et al. (2023) and D. C. Chu & Sun (2014), two key attitudinal domains—reactive and proactive—emerge from the studies, with both observed in the findings.

### Training and the Impact on Professional Practice

This review confirms existing research indicating that health and social care staff, police officers, and criminal justice professionals often lack the specialized training necessary to identify domestic abuse, resulting in frustration and inappropriate interventions (Christensen et al., 2021). The global gap in training is evident, with minimal undergraduate education on the topic and limited opportunities for ongoing professional development (Kirk & Bezzant, 2020). This lack of training diminishes professional competency in handling domestic abuse cases, while proactive responses, fueled by training, lead to increased empathy and fewer negative attitudes. Recent legislative changes have led to more police and criminal justice training; however, its effectiveness is contingent upon officers’ willingness to adapt the training beyond their personal views and organizational culture, as highlighted by Dempsey (2002) and Schein’s (1983) Model of Organizational Culture. There is a clear need for continuous, domestic abuse-specific professional development to ensure trauma-informed responses (A. Millar et al., 2022). A lack of professional efficacy contributes to disengagement, emphasizing the need for clear protocols and organizational support to improve proactive responses and enhance frontline professionals’ competence and confidence in addressing domestic abuse (Cleaver et al., 2019).

### Interpersonal Barriers Toward Professional Practice

Training alone does not fully explain reactive or proactive professional practice. Some healthcare professionals expressed concerns about making mistakes, such as incorrectly assuming someone is experiencing domestic abuse (Sundborg et al., 2017), while others hesitated to initiate conversations out of fear of offending or retraumatizing victims (Bradbury-Jones, 2015). These concerns are valid, as inappropriate responses can indeed lead to retraumatization (McLaughlin et al., 2024). Interpersonal barriers, such as a reluctance to pry or overstep, also contribute to disengagement, indirectly harming victims (Aregger Lundh et al., 2023; Murvartian, Saavedra-Macías, & Infanti, 2023). Previous research suggests that frontline professionals who acknowledge their responsibility in domestic abuse intervention tend to respond more empathetically and proactively (Roush & Kurth, 2016). Organizational and interpersonal barriers, such as heavy workloads and limited access to mental health resources, were also found to reduce sensitivity toward victim care, aligning with previous research (Maclean et al., 2024). These barriers can lead to desensitization, inadequate victim responses, systemic retraumatization, and negative outcomes for both victims and professionals.

### Psychosocial Factors and Cultural Norms

It is important to note that ratifying the Istanbul Convention (Council of Europe, 2024) does not necessarily mean that a country is effectively implementing the laws and policies it introduces. Therefore, an organization’s culture and its approach to addressing domestic abuse may not align with the country’s ratification status. The influence of organizational culture and street-level bureaucracy is evident in countries like Hungary, which has the highest rates of domestic abuse in the EU (Eurostat, 2024a). Despite this, Hungary has resisted adopting the Istanbul Convention, claiming its existing laws already protect women (European Parliament, 2020). However, Parti and Robinson (2021) found that Hungary’s criminal justice system does not prioritize victim needs and lacks standardized protocols. The focus on physical violence within these systems leads to a reactive professional approach, often misinterpreting coercive control and minimizing the impact of witnessing abuse on children (Elliffe & Holt, 2019; Myhill et al., 2023).

Psychosocial factors, such as sexism, gender stereotypes, and traditional beliefs, were common in the studies reviewed. These factors were associated with more negative attitudes toward domestic abuse, including victim-blaming and reactive practices. Research shows that gender roles and sexism predict tolerance of violence (Agadullina et al., 2022; Çalıkoglu et al., 2018), and male students in particular may justify abuse (Kerman & Ozturk, 2022). Gender-role attitudes, rather than gender alone, may shape perspectives on domestic abuse (Reidy et al., 2014).

Cultural differences, such as those found in cultures of honor in Turkey, Bosnia, and Hungary, may also contribute to reactivity. In these cultures, patriarchal standards emphasizing family reputation and male dominance hinder effective responses to abuse (Çalıkoglu et al., 2018; Tarhan et al., 2017). The review suggests that such organizational and cultural gender hierarchies influence professional practices, particularly in policing and criminal justice, where patriarchal norms impede domestic abuse responses (Carrillo, 2021). In contrast, healthcare systems, which often promote proactive, trauma-informed approaches and have more female representation, tend to offer more empathetic responses. However, acceptance and minimization of abuse were seen across all professions, indicating that professional practices are shaped by factors such as training availability, personal experiences, and the recognition of roles in addressing domestic abuse.

### Theoretical Implications for Practice

The theories of Lipsky’s (2010) street-level bureaucracy, Kahn et al.’s (1964) role stress theory, and Schein’s (1983) model of organizational culture provide valuable frameworks for interpreting the findings of the current review. Lipsky’s theory posits that public service workers, such as police officers and healthcare providers, possess significant discretion in their roles, influenced by personal experiences, judgments, and environmental factors. This aligns with the review’s findings, where frontline professionals’ responses to domestic abuse were shaped by individual experiences, training, and trauma-informed organizational structures. Kahn et al.’s (1964) Role Stress Theory highlights the stress professionals face when the demands on them exceed their available resources or capabilities. This pattern emerged in the review, where excessive workloads, understaffing, and inadequate funding led to frustration and reactive behaviors.

Finally, Schein’s (1983) model of organizational culture demonstrates how organizational values, beliefs, and behaviors shape professional practices. This was evident in the review, where health and social care professionals, influenced by trauma-informed organizational cultures, exhibited more proactive responses, while criminal justice and police professionals, shaped by different organizational values, tended to display more reactive behaviors. Together, these theories help explain the variability in professional responses to domestic abuse, as identified in the review.

### Implications for Research

This systematic review offers a novel perspective on the factors influencing professional practice in addressing domestic abuse, filling a gap in existing literature. It enhances understanding by examining how individual psychosocial factors, such as gender, empathy, sexism, and job role, interact with workplace factors and professional hierarchies to shape either proactive or reactive responses. The findings emphasize the importance of social ecology within the microsystem and mesosystem, highlighting how factors like gender, empathy, sexism, profession type, training, abuse experience, and country of origin contribute to disparities in professional responses. While distal influences, such as organizational policies, country-specific patriarchal or feminist ideologies, departmental resources, and bureaucratic barriers, were not fully explored, qualitative insights suggest these factors warrant further investigation (Clarke & Wydall, 2015; Gracia et al., 2014; Heffernan et al., 2014; Richards et al., 2021).

### Implications for Practice

The term “expert by experience” is often used for victims of domestic abuse, recognizing how their lived experience shapes policy and legislative development (Women’s Aid, 2024b). However, this term is not applicable to frontline professionals. This review introduces the concept of “novices by experience” to describe professionals who, through repeated exposure to domestic abuse, develop cynical, desensitized attitudes. Such mindsets often lead to victim-centered dehumanization or disengagement from the issue (Richards et al., 2021; Serrano-Montilla et al., 2023). These attitudes were compounded by a lack of multi-agency cohesion, where professionals frequently shifted responsibility to others, undermining effective intervention.

The review highlights key areas for improving professional training, particularly the need for clearly defined roles and responsibilities for each professional involved in addressing domestic abuse. Training should be tailored to the specific roles’ professionals hold within their organizations (Robinson, Myhill, et al., 2018). Interestingly, novice police officers were found to be more proactive in policing domestic abuse than their supervisory counterparts, who were more likely to minimize non-physical abuse and coercive control (Robinson, Myhill, et al., 2018). In contrast, healthcare and social care staff’s tolerance for domestic abuse was positively linked to their experience and staff ranking (Downie et al., 2019). This suggests that empathy is integral to addressing domestic abuse, with caregiving roles fostering a more empathetic response. Conversely, exposure to trauma in policing may diminish empathy, emphasizing the need for mandatory mental health support for police and criminal justice professionals. The review stresses that fostering proactive attitudes relies on timely, role-specific domestic abuse training and mental health support. It is recommended that all professionals receive targeted training early in their careers, with continuous support, to prevent the development of disengaged, late-career attitudes and ensure sustained empathetic practice.

### Implications for Policy

Not all studies included in this review were subject to the ratification of the Council of Europe’s (2024) Istanbul Convention. As ratification status varies, this review cannot fully clarify the specific roles and responsibilities of police officers, criminal justice practitioners, or healthcare professionals within their respective countries. Feedback from the studies suggests that organizational policies are often either non-existent or unknown to staff, highlighting the need for greater awareness and development of localized, organization-specific policies and procedures.

Ethical ambiguity in professional practice arises from policy deficiencies and undefined job roles, which undermine proactive interventions. Government and organizational policies should address these gaps by ensuring that funding, training, support, and resources are appropriately allocated to police, criminal justice, medical, and social care sectors. Furthermore, policies should encourage the development of a cohesive multi-agency approach, with clearly defined roles across professional bodies. The attitudes identified in this review emphasize the importance of these recommendations in enabling professionals to effectively handle domestic abuse cases, support victims, and enhance professional self-efficacy. Transparent roles and responsibilities within multi-agency coordination are critical for addressing organizational deficits and improving responses to domestic abuse. Table 3 presents a summary of the critical findings and recommendations.

**Table 3.** Summary of Critical Findings and Recommendations.

Research	1. Reactive practice is influenced by proximal factors in the micro and mesosystems such as profession type, sexism, empathy, and length of employment. 2. Further research into distal exosystem, macrosystem, and chronosystem is needed. 3. Standardized and psychometrically robust measures should be used to improve data quality.
Practice	1. This has bolstered the theoretical framework of reactive and proactive professional practice (Serrano-Montilla et al., 2023). 2. Pre-existing and preceding attitudes contributing to a negative professional practice have been highlighted. 3. Best practice must ensure that “at-risk” survivors’ experiences are not negated or minimized based on co-morbid mental health diagnoses or disabilities. 4. Professional roles and responsibilities must be clearly defined and transparent to organizations in the promotion of a coordinated multi-agency agenda.
Policy	1. Additional funds and resources must be provided to first responder and criminal justice authorities to improve domestic abuse training and practice. 2. A government and organization-wide policy must be developed to align professionals’ roles and responsibilities for the promotion of a multi-agency approach tackling reactive attitudes. 3. Staff members must undergo regular and up-to-date training, which must be adapted with changing domestic abuse legislation and expectations. 4. Staff members must also receive routine mental health support in the promotion of empathetic practice. 5. Staff must also be made aware of government and organization policy and their roles within the organization. 6. Organization-specific policy should be adapted for “at-risk” victim’s experiences of domestic abuse given their increased vulnerability.

### Limitations

This review has several limitations that should be acknowledged. Reflexivity plays a key role, as the researchers’ inclusion criteria and search strategies were influenced by their own knowledge and experiences with domestic abuse research. Coming from a victim-centered perspective, the researchers are particularly sensitive to the needs and experiences of those affected by violence. Furthermore, the researchers operate within a post-conflict society, where they have observed victim-perceived reactivity in frontline professionals, particularly in relation to domestic abuse cases. While the researchers aimed to conduct the research objectively, their personal experiences and understanding of systemic issues may have influenced the analysis, particularly with an emphasis on reactive responses from police, criminal justice, and social work professionals. The findings, which highlight patterns of reactivity in these sectors, reflect these insights, emphasizing a victim-centered approach.

Another limitation was the restriction of the search criteria to “knowledge, attitudes, and competencies,” which may have excluded relevant articles using alternative terms such as “myths,” “stigma,” or “beliefs,” potentially offering valuable insights. This limitation was evident when a general literature search revealed additional studies that would have been included had they appeared in the initial search. Future research could expand the search criteria to include a broader range of relevant studies (Murvartian et al., 2024; Roush & Kurth, 2016). Additionally, limiting the review to European published studies excluded relevant studies and grey literature from other regions, contributing to a limited pool of literature.

## Conclusion

With the introduction of the Istanbul Convention (Council of Europe, 2024), Europe has seen an increase in policies and protocols targeting domestic abuse and violence against women and girls. Previous European and international research has highlighted the inconsistent and contradictory professional attitudes, knowledge, and competencies regarding domestic abuse (Gracia et al., 2020). Gracia et al. (2020) called for further research to better understand the causes of these variations. This study expands on existing knowledge by identifying factors such as profession type, sexism, empathy, and training as key influences on professional attitudes toward domestic abuse within European frontline professionals (Gracia et al., 2020; Serrano-Montilla et al., 2023; Villagrán et al., 2024). However, a gap remains in understanding the complexities of domestic abuse among professionals, with organizational stigma and misinterpretation of abuse being central issues. The absence of consistent protocols has led to professionals downplaying the issue or shifting responsibility, rooted in ethical ambiguity. This review argues that domestic abuse should be considered as everyone’s responsibility, and policies should reflect this collective duty.
